# The role of health-related quality of life in risk prediction for developing cardiovascular disease, dementia and all-cause death among general older adults

**DOI:** 10.3389/fpubh.2022.1014019

**Published:** 2022-11-17

**Authors:** Aung Zaw Zaw Phyo, Joanne Ryan, Rosanne Freak-Poli

**Affiliations:** School of Public Health and Preventive Medicine, Monash University, Melbourne, VIC, Australia

**Keywords:** health-related quality of life, cardiovascular disease, dementia, cognitive decline, mortality, older people

The world is experiencing rapid growth in the aging population (1). Such a notable increase in human longevity is one of humanity's novel accomplishments of this century which reflects public health initiatives and healthcare improvement. In 2020, approximately 9% of the global population were aged ≥65 years representing 727 million and this proportion is expected to reach about 16% representing 2 billion in 2050 (1). On the other hand, aging also increases the risk of developing chronic age-related diseases such as diabetes, hypertension, osteoporosis, cardiovascular disease (CVD), and dementia (2). Among these age-related diseases, CVD and dementia are two of the leading causes of global disease burden, long-term disability claims and death in older people (3). Thus, the continued rapid growth in the aging population comes with an increased global disease burden, which places pressure on the healthcare resources as well as the society.

In this 21^st^ century, a patient-centric paradigm shift has occurred in healthcare and patient-centredness, which is respectful of an individual patient's values and preferences, is now widely considered a basic principle underpinning the provision of high-quality healthcare (4). Hence, the use of patient-reported outcome measures (PROMs) has gained increasing interest since they can capture not only an individual's perception of their health condition but also insights related to the improvements in functional capacity which are the ultimate goal of both healthcare providers and patients in seeking healthcare service (4).

The subjective health-related quality of life (HRQoL) is one of the most widely used PROMs in clinical research and public health surveillance. HRQoL consists of a broad multidimensional concept that includes physical, mental, emotional, and social health functioning domains (5). It captures the impact of an individual's health condition on their own daily activities (5). Therefore, together with the current patient-centric movement, HRQoL has received increasing attention in healthcare settings, particularly as a patient's HRQoL can be monitored to evaluate the recovery progress of the patient following a specific medical treatment such as surgery (4). Further, there is evidence that

lower HRQoL predicts worse prognosis, rehospitalization, and overall survival in people with chronic diseases including CVD (6, 7). Thus, HRQoL has been considered a novel prognostic indicator in the patient population, including among CVD patients.

In the context of non-clinical settings, we recently conducted the prospective analyses using five-year longitudinal data of the ASPREE (ASPirin in Reducing Events in the Elderly) cohort including 19,106 community-dwelling individuals aged ≥70 years (all others) or ≥65 years (United States minority i.e., African American and Hispanic groups only) who had no prior CVD events, diagnosed dementia or persistent physical disability at the time of study entry (8–11). Therefore, our ASPREE study participants who were “free” of major health events at baseline could be employed to address the predictive value of HRQoL for the development of chronic health outcomes. Furthermore, our study cohort had a good diversity in gender (56.4%, women), educational level (54.8%, ≥12 years of education), and baseline health-related behaviors (44.7%, former/current smoker; 52.8%, current low-risk alcohol consumption) (8–10). Although our unique ASPREE cohort was initially “healthy” at the time of study entry, the study participants also had other chronic diseases such as hypertension (74.3%) and dyslipidaemia (65.2%) which are usually found in community-dwelling older adults (8–10). Therefore, this sample could perhaps be representative of relatively “healthy” community-dwelling older people in primary care settings. In Australia, the postcode for the area of residence was additionally recorded and we estimated a macro-level socioeconomic position (Socio-Economic Indexes for Areas Index of Relative Socio-economic Advantage and Disadvantage (SEIFA-IRSAD), divided into quintiles) (12). Our Australian sample, which represents 87.4% of the cohort overall, was evenly distributed across all socioeconomic quintiles (12). In our prospective analyses, we found that the self-reported 12-item short form HRQoL assessment (SF-12, version-2) can predict the future risk of CVD, cognitive decline, dementia, and all-cause mortality in older adults, even when adjusting for sociodemographic factors, health-related behaviors and clinical measures (8–10). Specifically, over a median of 4.7 years, a 10-unit higher physical HRQoL at baseline was associated with a lower risk of incident CVD by 14%, cognitive decline by 6%, and all-cause mortality by 17% (Figure 1) (8–10). A 10-unit higher mental HRQoL was associated, with a 12% lower risk of cognitive decline and 15% lower risk of dementia, but was not associated with incident CVD or all-cause mortality (Figure 1) (8–10). Next, we assessed whether changes in physical HRQoL (i.e. trajectories) over a median of 4.7 years among the same ASPREE cohort (11). We found that older individuals in the declining physical HRQoL trajectory (i.e., about 9 units decrease over a median of 4.7 years follow-up) had approximately 50% higher subsequent risk of incident CVD in the next 2 years compared to those in the high physical HRQoL trajectory group (11).

**Figure 1:**
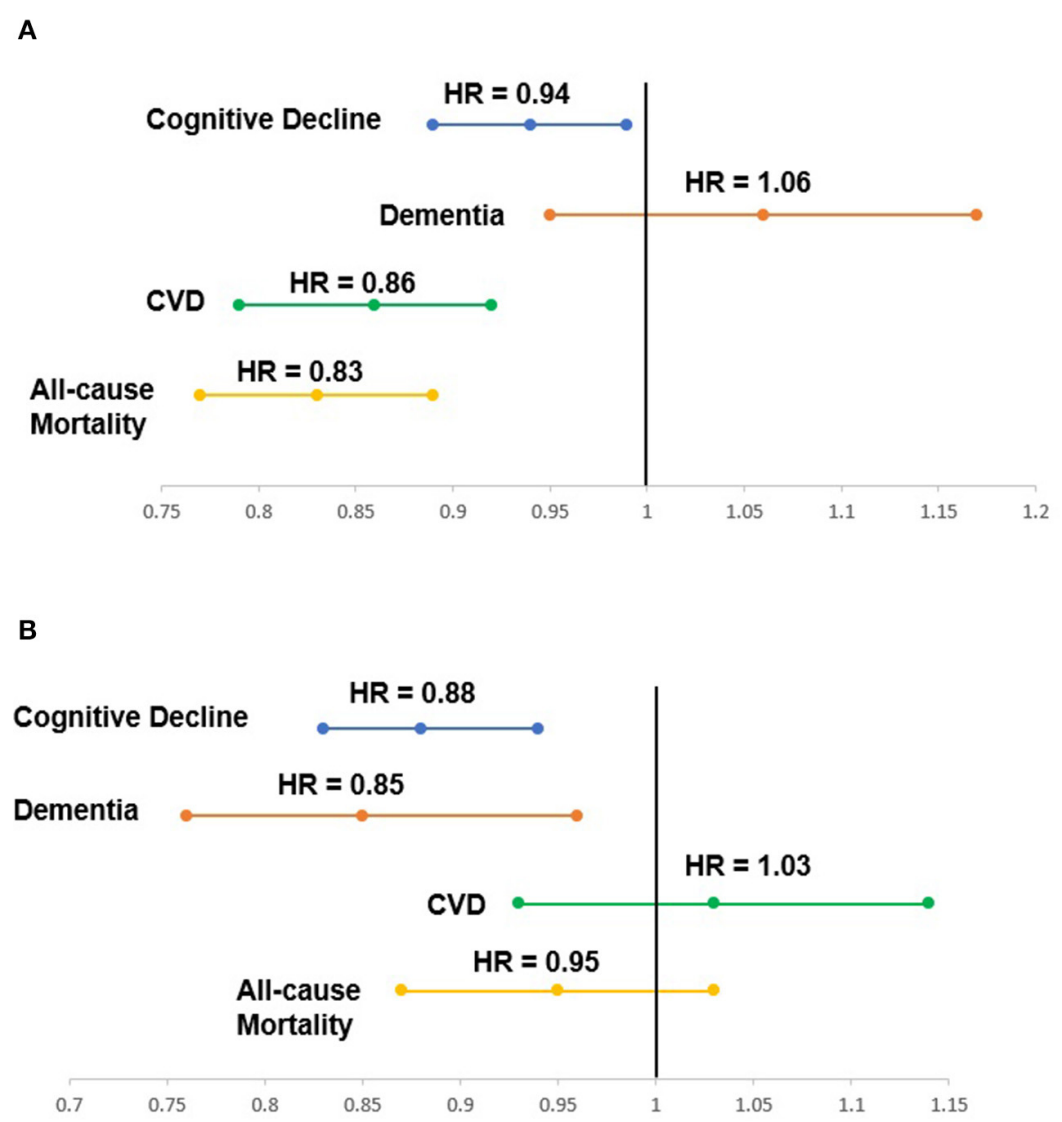
**(A)** Association between physical health-related quality of life at baseline and the risk of health outcomes over a median of 4.7 years; **(B)** Association between mental health-related quality of life at baseline and the risk of health outcomes over a median of 4.7 years.

In this manner, poor HRQoL has also been shown to increase the risks of adverse health outcomes in community-dwelling older adults. These recent findings reflect the importance of an individual's perception of their health status (i.e., HRQoL) in primary care settings. Additionally, our findings support the Australian Commission on Safety and Quality in Health Care's government recommendations that PROMs, including HRQoL, be incorporated into the health system as a policy goal (13). One limitation for the integration of the HRQoL measure into the routine clinical practice is how to score the HRQoL as the HRQoL has a multi-step calculation to derive the summarized component scores of physical and mental HRQoL. Thus, future efforts should be made on developing an automatic scoring system, providing a superior option for clinicians to implement routine HRQoL measurements in primary care settings.

Notably, our findings are independent of traditional risk factors for developing age-related diseases such as age, gender, lifestyle factors, co-morbidities, and objective clinical measures - body mass index, blood pressure and total cholesterol level, which are all included in existing risk prediction models. This demonstrates that in addition to our known modifiable and non-modifiable risk factors, HRQoL can predict future risk of CVD, cognitive decline, dementia and all-cause death in community-dwelling older people. To the best of our knowledge, an individual's subjective perception of health status is yet to be incorporated into disease risk scores. Our preliminary findings suggest that there is considerable potential to expand the use of an inexpensive HRQoL measure in chronic disease risk assessments, especially for older people. In this approach, earlier identification of older individuals at a higher risk of chronic diseases could increase the efficacy of healthcare resources for the aging population by focusing primary prevention on those who need it the most. Overall, based on our emerging evidence and prior research, we, as HRQoL researchers, call for healthcare professionals to appraise the insight of an individual's perception of their health conditions (i.e., self-reported HRQoL) in the risk assessment for developing CVD, dementia, and all-cause deaths among general older people in this patient-centric era.

## Author contributions

AZZP wrote the initial manuscript draft and undertook revisions. JR and RF-P provided critical comments and suggestions. All authors approved the final version.

## Funding

AZZP was supported by Monash University Postgraduate Publications Award, Monash International Tuition Scholarship and Monash Graduate Scholarship (30072360). JR is supported by a National Health and Medical Research Council Dementia Research Leader Fellowship (1135727). RF-P is supported by a National Heart Foundation of Australia Postdoctoral Fellowship (101927). Funders had no role in the design and conduct of the study, in the writing, and submission of the manuscript.

## Conflict of interest

The authors declare that the research was conducted in the absence of any commercial or financial relationships that could be construed as a potential conflict of interest.

## Publisher's note

All claims expressed in this article are solely those of the authors and do not necessarily represent those of their affiliated organizations, or those of the publisher, the editors and the reviewers. Any product that may be evaluated in this article, or claim that may be made by its manufacturer, is not guaranteed or endorsed by the publisher.
